# MAGI2-AS3 restrains proliferation, glycolysis, and triggers apoptosis in acute lymphoblastic leukemia via regulating miR-452-5p/FOXN3 pathway 

**DOI:** 10.22038/IJBMS.2021.58963.13095

**Published:** 2022-01

**Authors:** Xiao-Guang Chen, Bing-Hua Dou, Jin-Dou An, Song Feng, Na Liu, Guang-Yao Sheng

**Affiliations:** 1 Department of Pediatrics, The First Affiliated Hospital of Zhengzhou University, Zhengzhou 450052, Henan Province, P.R. China

**Keywords:** ALL, Apoptosis, FOXN3, Glycolysis, Growth, MAGI2-AS3, miR-452-5p

## Abstract

**Objective(s):**

MAGI2-AS3 is a cancer suppressor gene of multiple malignancies. Acute lymphoblastic leukemia (ALL) is an important type of leukemia that especially occurs in children. Our work evaluated the modulation of MAGI2-AS3 in ALL.

**Materials and Methods:**

qPCR and Western blotting were adopted for detection of target molecular expression. Growth and apoptosis were determined by CCK8 assay and Annexin V/PI staining. Glycolysis was detected by commercial kits. The direct binding between miR-452-5p and MAGI2-AS3 or FOXN3 was assessed by luciferase reporter assay. Tumor growth was measured in nude mice *in vivo*.

**Results:**

MAGI2-AS3 was down-regulated in ALL. Enforced expression of MAGI2-AS3 inhibited growth and glycolysis while promoting apoptosis of ALL cells. Moreover, MAGI2-AS3 up-regulated FOXN3 via sponging miR-452-5p. FOXN3 depletion abrogated MAGI2-AS3-mediated anti-cancer action. More importantly, MAGI2-AS3 repressed ALL cell growth in nude mice through regulation of miR-452-5p/FOXN3.

**Conclusion:**

MAGI2-AS3 inhibits ALL development via modulating miR-452-5p/FOXN3.

## Introduction

Acute lymphoblastic leukemia (ALL) is an important hematologic malignancy commonly occurring in children (1). ALL can be subdivided into B-cell ALL and T-cell ALL based on immunophenotypes. In spite of improvement in survival, ALL patients with a second relapse still have bad outcomes (2). Besides, toxic side effects during chemotherapy may result in the occurrence of secondary cancer (3). Therefore, better understanding the pathological mechanisms of ALL can provide new treatment interventions for ALL. 

Long non-coding RNAs (lncRNAs) without protein-coding abilities are composed of more than 200 nucleotides (4). LncRNAs are proven to participate in occurrence and progression of multiple tumors (5, 6). It has been revealed that lncRNAs act as regulators during occurrence, development, or relapse of cancer (7, 8). In addition, dysregulation of lncRNAs participates in ALL progression (9). MAGI2-AS3 level is declined, which promotes progression of various tumors, including breast carcinoma, lung carcinoma, liver cancer, and bladder tumor (10). It is worth noting that MAGI2-AS3 level was reduced in ALL clinical specimens (11). It has been documented that low expression of MAGI2-AS3 in acute myeloid leukemia facilitated the stemness of tumor stem cells (12). However, whether MAGI2-AS3 can be involved in ALL progression has not been reported. 

MiRNAs are another type of non-coding RNAs, post-transcriptionally modulating gene expression (13). miRNAs are involved in regulation of different pathophysiological processes, such as malignancy (14). MiR-452-5p was reported to facilitate tumorigenesis among various cancers. For example, high miR-452-5p level contributed to colorectal carcinoma progression through regulating PKN2/ERK/MAPK (15). For lung cancer, a higher miR-452-5p level promoted cancer development by regulating CDKN1B (16). Nevertheless, the involvement of miR-452-5p in ALL remains to be clarified. 

Forkhead box N3 (FOXN3) belongs to the forkhead box family, which takes part in multiple biological processes, such as tumor development (17). Down-regulation of FOXN3 has been found in different carcinomas, such as ALL (18, 19). Interestingly, miR-452-5p was predicted to directly bind to either MAGI2-AS3 or FOXN3. Thus, we speculated that MAGI2-AS3 might affect ALL development via miR-452-5p/FOXN3. 

The current work elucidated the modulation of MAGI2-AS3 in ALL progression. Our data may provide a theoretical basis for developing novel interventions for ALL. 

## Materials and Methods

***Sample collection***

Bone marrow samples were obtained from 25 ALL cases and normal volunteers with their signed informed consent. This study in accordance with the Declaration of Helsinki was approved by the Ethics Committee. 

***Cell culture and transfection***

Human ALL cells were purchased from the Cell Bank of the Chinese Academy of Sciences (Shanghai, China) and maintained in RPMI-1640 medium (Thermo Fisher, USA) containing 10% FBS (Biological Industries, Israel). PBMCs were isolated from the serum of healthy volunteers and maintained in RPMI-1640 medium with 10% FBS. 

MAGI2-AS3 expression plasmid, vector plasmid, miR-452-5p mimics, and OXN3 shRNA (shFOXN3) were purchased from GenePharma (Shanghai, China). The above segments were transfected into ALL cells using Lipofectamine 2000 (Thermo Fisher).

***Quantitative polymerase chain reaction (qPCR)***

Total RNA was isolated from ALL cells using TRIzol solution (Thermo Fisher). Subsequently, cDNA was synthesized using a Prime Script RT kit (Takara, Japan). The SYBR-Green PCR Master Mix (Applied Biosystems, USA) was used for qPCR. The relative levels of genes were calculated with the 2^−ΔΔCt^ method. 

***Cell counting kit-8 (CCK-8)***

Cells were incubated with 10 μl of CCK-8 solution (Dojindo, Japan) at 37 °C for different time periods. After reaction for 4 hr, the results were measured at 450 nm using a microplate reader (Tecan, Switzerland).

***Apoptosis detection***

To evaluate apoptosis, the collected cells were stained with Annexin V and PI for 30 min away from light. The cells were then assessed using a flow cytometer (Millipore, USA). 

***Glycolysis determination***

The glycolysis of ALL cells was determined by detecting glucose uptake, lactate, and ATP level using commercial kits according to the manufacturers’ instructions. 

***Western blotting***

Total protein was prepared using the Pierce IP lysis buffer (Thermo Fisher) and quantified with a BCA kit (Thermo Fisher). Thereafter, the protein was separated by SDS-PAGE and blotted onto PVDF membranes. After blocking with 5% skim milk, the membranes were probed with primary antibodies against PCNA (1:1000, Abcam), Bcl-2 (1:1000, Abcam), Bax (1:1000, Abcam), HK2 (1:1000, Abcam), FOXN3 (1:100, Thermo Fisher), and GAPDH (1:1000, CST, USA) at 4 °C overnight. Then, a secondary antibody (1:4000, Thermo Fisher) was used. The protein bands were visualized using the enhanced chemiluminescence (ECL) (Thermo Fisher). 

***Dual-luciferase reporter assay***

The wild-type (WT) or mutant (MUT) sequences of MAGI2-AS3 or FOXN3 3’UTR containing miR-452-5p binding sites were subcloned into the psiCHECK vector. Subsequently, cells were transfected with WT or MUT plasmid and miR-NC or miR-452-5p mimics using Lipofectamine 2000. After 48 hr, a Dual-Lucy Assay Kit (Solarbio) was used to assess luciferase activities. 

***Xenograft model***

The BALB/c nude mice (18–22 g) were purchased from Shanghai SLAC Laboratory Animal Co., Ltd. The mice were injected with stably transfected Jurkat cells (1 × 10^7^). Tumor length and width were measured every 72 hr. One month later, tumors were collected, weighed, and detected by the following experiments. All experimental protocols were approved by the Animal Ethics Committee.

***TUNEL***

The apoptosis was detected using the Colorimetric TUNEL Apoptosis Assay Kit (Beyotime). In brief, the tumors were subjected to paraffin embedding and sectioned. The 5-μm sections received deparaffination, hydration, and antigen retrieval. After treatment with Proteinase K and blocking in 3% H_2_O_2_ for 20 min, the reaction was performed at 37 °C for 1 hr, and then reaction with streptavidin-HRP and DAB solution. Finally, the sections were examined using an inversion microscope.

***Statistical analysis***

Data are expressed as mean± standard deviation (SD). Student’s t-test for two-group or one-way ANOVA for multiple group comparison was performed using GraphPad Prism 6.0. P<0.05 was considered statistically significant.

## Results

***MAGI2-AS3 was up-regulated in ALL***

**Figure 1:**
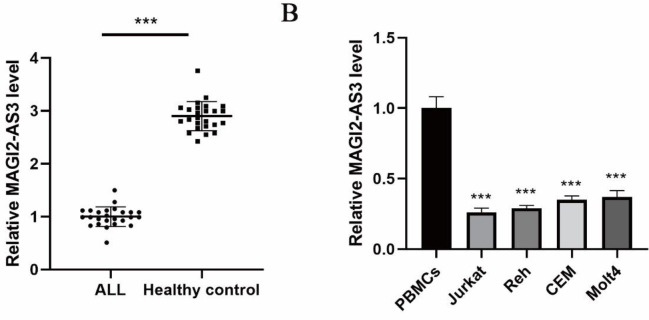
Increased expression of MAGI2-AS3 in ALL. (A) MAGI2-AS3 levels in ALL cases and normal controls were assessed by qPCR. (B) MAGI2-AS3 expression among various ALL cell lines was detected by qPCR. *** *P*<0.001

The aberrant level of MAGI2-AS3 in clinical samples of ALL cases and normal controls was detected by qPCR. We observed a lower level of MAGI2-AS3 in the ALL group as compared with the control group (Figure 1A). Consistent with this, MAGI2-AS3 level was declined in different tumor cell lines as compared with PBMCs (Figure 1B). Therefore, down-regulation of MAGI2-AS3 might play pivotal roles during the malignant development of ALL.

***MAGI2-AS3 restrained proliferation, glycolysis, and induced apoptosis in ALL cells***

**Figure 2:**
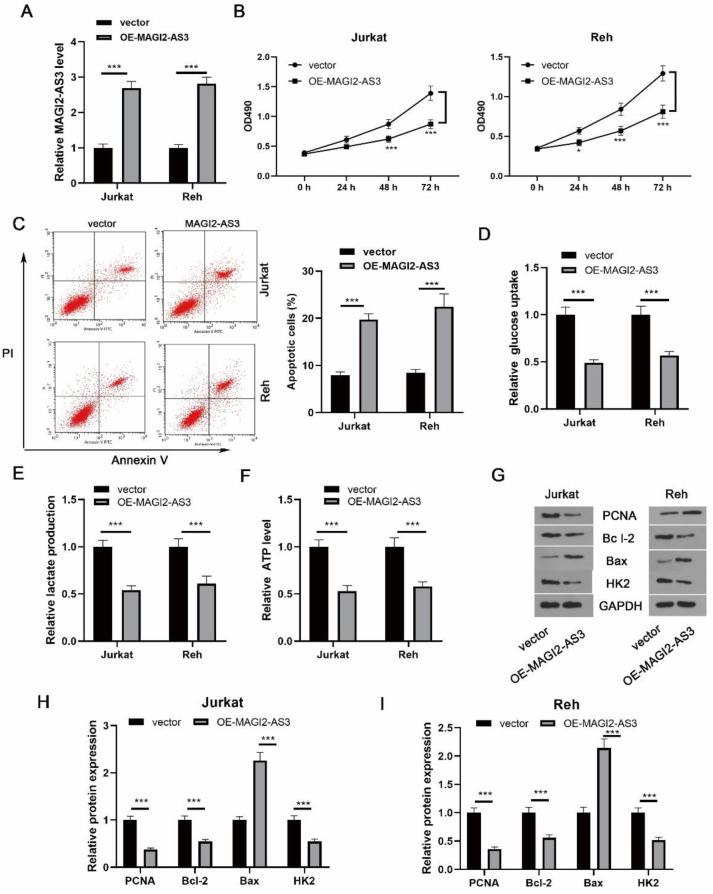
Effect of MAGI2-AS3 on growth, apoptosis, and glycolysis of ALL cells. (A) qPCR for determination of overexpression efficiency of MAGI2-AS3. (B) Cell growth was determined by CCK-8. (C) Apoptosis was measured by Annexin V/PI staining. The glucose uptake (D), lactate production (E), and ATP level (F) were measured. (G-I) Protein expression was assessed by Western blotting. * *P*<0.05 and *** *P*<0.001

First, the OE-MAGI2-AS3 plasmid was transfected into ALL cells to evaluate its biological function. The overexpression efficiency of MAGI2-AS3 was validated (Figure 2A). The growth of ALL cells was remarkably repressed after MAGI2-AS overexpression (Figure 2B). Moreover, MAGI2-AS3 overexpression significantly enhanced the apoptotic rate of ALL cells (Figure 2C). It is accepted that the Warburg effect contributes to tumor development(20). Figures 2D-F demonstrated that enforced expression of MAGI2-AS3 promoted glucose uptake, lactate, and ATP release. Moreover, Western blotting analysis showed that PCNA, Bcl-2, and HK2 were down-regulated, but Bax was up-regulated in MAGI2-AS3-overexpressed ALL cells (Figure 2G-I). The above observations revealed that MAGI2-AS3 repressed growth, glycolysis and triggered apoptosis of ALL cells. 

***MAGI2-AS3 increased FOXN3 level via sequestering miR-452-5p***

**Figure 3:**
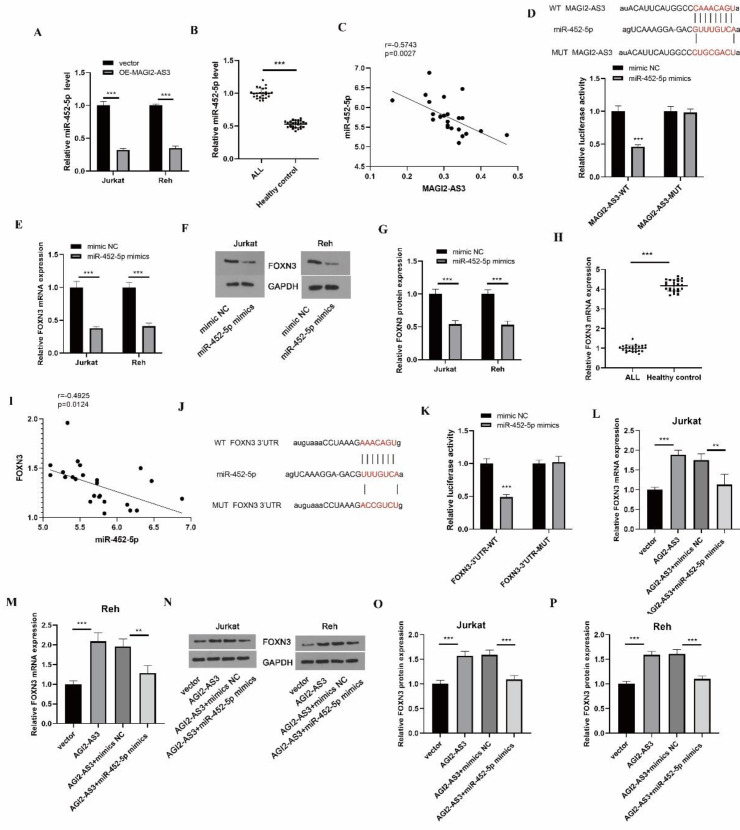
MAGI2-AS3 sponges miR-452-5p to promote FOXN3 expression. (A) MiR-452-5p level in MAGI2-AS3-overexpressed cells was assessed by qPCR. (B) The expression level of miR-452-5p in ALL cases and healthy controls was evaluated by qPCR. (C) Correlation between MAGI2-AS3 expression and miR-452-5p level in ALL samples. (D) Dual-luciferase reporter assay was performed to detect the interaction between MAGI2-AS3 and miR-452-5p. FOXN3 expression was detected by qPCR. (E) and Western blotting (F-G). (H) FOXN3 mRNA level in clinical specimens was determined by qPCR. (I) Correlation between FOXN3 and miR-452-5p level in ALL specimens. (J) The Starbase database predicted the binding sites of miR-452-5p in FOXN3. (K) The direct binding between FOXN3 and miR-452-5p was confirmed by dual-luciferase reporter assay. FOXN3 level was detected by qPCR (L-M) and Western blotting (N-P). * *P*<0.05, ** *P*<0.01, and *** *P*<0.001

Next, the downstream modulatory mechanisms of MAGI2-AS3 were elucidated. The miR-452-5p level was reduced by MAGI2-AS3 overexpression (Figure 3A). Besides, a high expression of miR-452-5p was observed in ALL specimens (Figure 3B). MAGI2-AS3 was negatively associated with miR-452-5p expression (Figure 3C). Moreover, the predicted binding between MAGI2-AS3 and miR-452-5p was verified (Figure 3D). Furthermore, expression of FOXN3 was declined after transfection with miR-452-5p mimics (Figures 3E-G). Besides, a high expression of FOXN3 was verified in ALL (Figure 3H) and was negatively associated with miR-452-5p expression (Figure 3I). The Starbase database speculated that FOXN3 could bind to miR-452-5p (Figure 3J). miR-452-5p mimics transfection remarkably decreased the luciferase activity in the FOXN3 WT group, whereas there was no distinct change in the FOXN3-3’UTR MUT group (Figure 3K). Additionally, enforced expression of MAGI2-AS3 raised FOXN3 levels, which could be reversed by miR-452-5p mimics (Figures 3L-P). Taken together, MAGI2-AS3 overexpression enhanced FOXN3 level by sequestering miR-452-5p. 

***FOXN3 depletion abolished the anti-tumor action of MAGI2-AS3***

**Figure 4:**
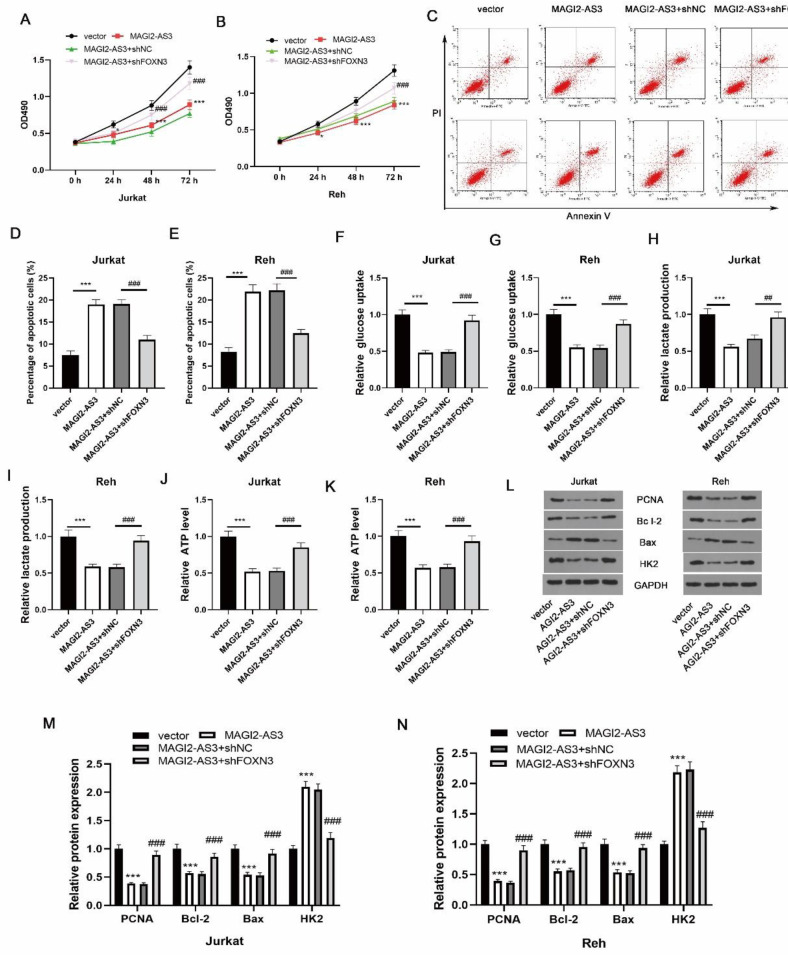
FOXN3 depletion abrogates the anti-tumor action of MAGI2-AS3. (A-B) Proliferative ability was assessed by CCK8 assay. (C-E) Annexin V/PI staining was adopted for determination of apoptosis. (F-K) Glycolysis was assessed by commercial kits. (L-N) Western blotting for target protein expression. * *P*<0.05 and *** *P*<0.001; ## *P*<0.01 and ### *P*<0.001 versus the indicated group

To explore whether MAGI2-AS3 affected ALL development through regulating FOXN3, the MAGI2-AS3-overexpressed cells were further transfected with shFOXN3. Functional assay suggested that the inhibited growth ability in MAGI2-AS3 overexpressed cells was recovered by FOXN3 depletion (Figures 4A&B). Furthermore, FOXN3 knockdown restrained MAGI2-AS3-triggered apoptosis (Figures 4C-E). FOXN3 silencing promoted glycolysis in MAGI2-AS3-overexpressed cells (Figures 4F-K). Additionally, MAGI2-AS3 overexpression reduced PCNA, Bcl-2, and HK2 levels, and enhanced the Bax level, however, their changes could be reversed by shFOXN3 (Figures 4L-N). Collectively, MAGI2-AS3 overexpression inhibited ALL malignant progression through regulating FOXN3. 

***MAGI2-AS3 overexpression restrained tumor growth in vivo***

**Figure 5:**
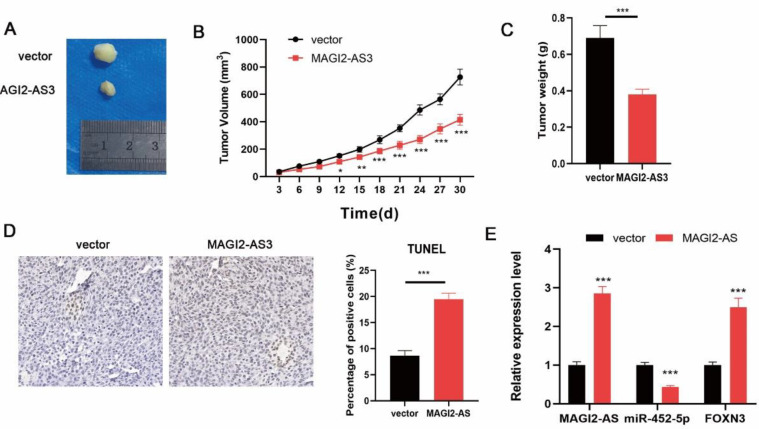
MAGI2-AS3 depletion repressed tumor growth in vivo. (A) Tumors were collected and photographed. Tumor volume (B) and weight (C) were assessed. (D) TUNEL assay for evaluating apoptosis in tumors. (E) MAGI2-AS3, FOXN3, and miR-452-5p expression in tumors were measured by qPCR. * *P*<0.05, ** *P*<0.01, and *** *P*<0.001

Finally, the effect of MAGI2-AS3 on tumorigenesis *in vivo* was evaluated. After MAGI2-AS3 overexpression, the tumor volume and weight were obviously declined. In addition, apoptosis in tumors was promoted by MAGI2-AS3 (Figure 5D). Moreover, MAGI2-AS3 overexpression resulted in increased MAGI2-AS3 and FOXN3 levels but decreased miR-452-5p expression in tumors (Figure 5E). Collectively, MAGI2-AS3 repressed tumorigenesis in vivo by affecting the miR-452-5p/FOXN3 pathway. 

## Discussion

Huge improvements have been achieved in uncovering the pathological mechanisms of ALL during the past few years. Nevertheless, the exact mechanisms of ALL cell proliferation, metastasis, and recurrence are still undefined. The current work elucidated a new MAGI2-AS3/miR-452-5p/FOXN3 axis in ALL malignant progression. Our data showed that MAGI2-AS3 and FOXN3 were highly expressed, while miR-345-5p was lowly expressed in ALL. Mechanistically, MAGI2-AS3 sponged miR-345-5p to increase the FOXN3 level, conferring malignant properties of ALL cells.

MAGI2-AS3, situated at chromosome 7q21.11, has been documented to regulate the development of various tumors (21). MAGI2-AS3 modulates the malignant capacities of malignancies in different ways, including the ceRNAs mechanism. For example, MAGI2-AS3 favored colorectal carcinoma abnormal proliferation and metastasis via targeting miR-3163(22). MAGI2-AS3 was reported to increase MAGI2 expression by inhibiting MAGI2 demethylation, which restrained growth and metastasis of breast carcinoma (23). Moreover, MAGI2-AS3 delayed the malignant progression of esophageal tumors via transcriptional repressing HOXB7 expression (24). In a recent study, MAGI2-AS3 exerted a carcinogenic effect in acute myeloid leukemia (12). In our study, the MAGI2-AS3 level was declined and restoration of its expression repressed malignant capacities in ALL. Therefore, these results firstly suggest that MAGI2-AS3 acted as a cancer suppressor gene to delay ALL progression.

Glucose metabolic reprogramming is an important feature of malignancies (25). Unlike normal cells, cancer cells prefer to alter energy metabolism via glycolysis, causing increased glucose uptake and lactate release (26). Glycolysis is a special phenomenon under normal conditions, cancer cells still produce ATP to provide energy for tumor cells via glycolysis (27). Glycolysis contributed to development and lowered drug sensitivity of ALL cells(28). HK2 is a specific protein that catalyzes glycolysis (29). Cancer cells have a sensitive response to glycolysis suppression and targeting the glucose metabolic pathway is considered a novel strategy for cancer therapy (30). The current study proved that glycolysis was repressed, also the HK2 level was declined after MAGI2-AS3 overexpression. Thus, MAGI2-AS3 inhibited ALL progression via repressing glycolysis. 

Cancer cells must balance the energy metabolic requirements of rapid cellular proliferation to maintain their survival (31). It has been verified that glycolysis confers proliferation, apoptosis resistance, metastasis, immune escape, and drug resistance of cancer cells (32). Given all this, the malignant abilities of ALL cells were further determined. As expected, MAGI2-AS3 effectively inhibited the malignant capacities of ALL cells. PCNA is a cofactor of DNA polymerase δ, which takes part in DNA replication and has been recognized as a marker to evaluate tumor cell growth (33). The balance between proliferation and apoptosis is regulated by the Bcl-2 family containing pro-apoptotic protein Bax and anti-apoptotic protein Bcl-2 (34). In the current study, MAGI2-AS3 overexpression significantly cut down PCNA and Bcl-2 expression but promoted Bax expression in ALL cells. These data indicated that inhibition of glycolysis by MAGI2-AS3 may further repress the malignancy of ALL. 

We next probed the downstream pathway of MAGI2-AS3 during ALL development. The ceRNA mechanism is that lncRNAs located in cytoplasm adsorb miRNAs to raise the expression of miRNA targets. The involvement of miR-452-5p in carcinomas has been widely documented. For instance, miR-452-5p could contribute to colorectal cancer progression through activating ERK/MAPK feedback loop (15). MiR-452-5p accelerated the carcinogenesis of hepatocellular carcinoma (35). Growing evidence has proven the interplay between miR-452-5p and lncRNA. SOX2-OT accelerated the progression of prostate carcinoma by reducing the miR-452-5p level (36). LINC00052 sponged miR-452-5p to reverse the malignant capacities of live cancer cells (37). However, we have no idea about the regulator roles of miR-452-5p in ALL. Importantly, we demonstrated that miR-452-5p was up-regulated, and negatively associated with MAGI2-AS3 expression in ALL specimens. Furthermore, dual luciferase assay confirmed the interaction between MAGI2-AS3 and miR-452-5p. Collectively, reduction of MAGI2-AS3 expression caused an aberrant increase in miR-452-5p level, which accelerated malignant development of ALL. 

For exploration of the downstream target of the MAGI2-AS3/miR-452-5p axis in ALL, FOXN3 was selected due to its potential binding to miR-452-5p. FOXN3 repressed the development of several cancers. FOXN3 repressed the development of colorectal malignancy through inactivation of β-catenin/TCF (38). FOXN3 played a tumor inhibitory role in papillary thyroid carcinoma via inactivating the Wnt/β-catenin pathway (39). Interestingly, FOXN3 was confirmed to be down-regulated in ALL (19). However, whether the MAGI2-AS3/miR-452-5p pathway exerted a regulatory effect on ALL cells via targeting FOXN3 remains unclear. Consistently, we indicated that FOXN3 expression was reduced in ALL. Additionally, FOXN3 was proven to be a target of miR-452-5p. More importantly, MAGI2-AS3 overexpression led to a significant reduction in FOXN3 expression, which was counteracted by miR-452-5p overexpression. MAGI2-AS3-mediated inhibition in the malignant phenotypes of ALL cells was abolished by FOXN3 knockdown. Taken together, MAGI2-AS3/miR-452-5p/FOXN3 pathway participated in the pathological mechanisms of ALL.

## Conclusion

MAGI2-AS3 functioned as a cancer suppressor and enforced expression of MAGI2-AS3 inhibited growth, glycolysis, and triggered apoptosis of ALL cells by sponging miR-452-5p to enhance FOXN3 expression. Our findings help to uncover ALL pathogenesis and provide evidence for MAGI2-AS3 as an effective intervention for treating ALL.

## Authors’ Contributions

XGC and GYS Study conception and design; XGC, BHD, JDA SF, and NL Data analysis and draft manuscript preparation; GYS Critical revision of the paper; GYS Supervision of the research; XGC, BHD, JDA SF, NL, and GYS Final approval of the version to be published.

## Conflicts of Interest

The authors declare that no conflict of interest exists.
